# S100 Calcium‐Binding Protein 16 (S100A16) Promotes Hepatic Stellate Cell Activation Via Erb‐B2 Receptor Tyrosine Kinase 2 (ERBB2)‐mediated Glycolysis to Facilitate Liver Fibrosis

**DOI:** 10.1002/cbf.70299

**Published:** 2026-09-07

**Authors:** Shiyu Ou, Xiaoling Tang, Juan Liu, Rong Ouyang, Shijiang Huang, Shuwen Weng, Jinping Qin, Dingheng Liang, Ling Du, Zhongzhuan Li

**Affiliations:** ^1^ Department of Gastroenterology Liuzhou Workers' Hospital (The Fourth Affiliated Hospital of Guangxi Medical University) Liuzhou P. R. China; ^2^ Department of Pharmacy Guangxi Zhuang Autonomous Region Brain Hospital Liuzhou P. R. China

**Keywords:** ERBB2, glycolysis, hepatic stellate cell activation, liver fibrosis, S100A16

## Abstract

Hepatic stellate cell (HSC) activation is a key mediator in hepatic fibrosis, with glycolysis serving a key regulatory function. S100 calcium‐binding protein 16 (S100A16) has been associated with various pathologies; however, its function and underlying mechanisms in liver fibrosis remains elusive. This study aimed to elucidate the functional role of S100A16 in HSC activation and its interaction with Erb‐B2 receptor tyrosine kinase 2 (ERBB2)‐mediated glycolysis. Transforming growth factor‐β1 (TGF‐β1)‐stimulated human liver stellate cells (LX2) and carbon tetrachloride (CCl_4_)‐induced mice were used as in vitro and in vivo models, respectively. Cell proliferation and alpha‐smooth muscle actin (α‐SMA) expression were detected by Cell Counting Kit‐8 (CCK‐8) assay and immunofluorescence. Glycolysis was detected via measuring the extracellular acidification rate (ECAR), lactate production, glucose uptake, and glycolytic enzyme levels. Molecular interactions were analyzed through bioinformatics and co‐immunoprecipitation. Liver injury and fibrosis were assessed using hematoxylin and eosin (H&E)/Masson staining. Expression of S100A16, ERBB2, and fibrosis markers were quantified using quantitative real‐time PCR (qRT‐PCR) and Western blot. S100A16 was upregulated in fibrotic liver tissues and TGF‐β1‐stimulated LX2 cells. TGF‐β1 increased lactate production and glycolysis, whereas S100A16 knockdown produced the opposite effects. Additionally, TGF‐β1 enhanced cell proliferation and upregulated α‐SMA, Collagen 1, and Fibronectin levels, but these effects were attenuated by either S100A16 knockdown or glycolysis inhibition. S100A16 interacted with ERBB2 and promoted its expression. ERBB2 knockdown reduced TGF‐β1‐induced glycolysis and HSC activation, along with reduced cell proliferation, effects that were inhibited by S100A16 overexpression. Furthermore, S100A16 knockdown ameliorated CCl_4_‐induced liver injury and fibrosis, decreased serum lactate levels, and reduced S100A16 and ERBB2 expression, thereby suppressing glycolysis. Collectively, S100A16 promotes HSC activation and liver fibrosis through ERBB2‐mediated glycolysis, highlighting the S100A16‐ERBB2‐glycolysis axis as a potential therapeutic target.

## Abstract 2

**Significance Statement:** This study identifies the S100A16‐ERBB2‐glycolysis axis as a novel driver of HSC activation. Targeting this metabolic checkpoint may offer a new therapeutic strategy for liver fibrosis.

## Highlights

- S100 calcium‐binding protein 16 (S100A16) promotes the activation of hepatic stellate cells via inducing glycolysis.
- S100A16 interacts with Erb‐B2 receptor tyrosine kinase 2 (ERBB2) to promote its expression.
- S100A16 promotes hepatic stellate cell activation and liver fibrosis via ERBB2‐mediated glycolysis.

**Abbreviations**
2‐DG: 2‐deoxy‐D‐glucose
ALT: Alanine aminotransferase
AST: Aspartate aminotransferase
CCK‐8: Cell Counting Kit‐8
CCl_4_
: Carbon tetrachloride
Co‐IP: co‐immunoprecipitation
DMEM: Dulbecco's modified Eagle's medium
ECAR: Extracellular acidification rate
ECM: Extracellular matrix
ERBB2: Erb‐B2 receptor tyrosine kinase 2
FBS: Fetal bovine serum
HK2: Hexokinase II
HSCs: Hepatic stellate cells
H&E: Hematoxylin and eosin
LDHA: Lactate dehydrogenase A
qRT‐PCR: Quantitative real‐time PCR
S100A16: S100 calcium‐binding protein 16
shRNA: Short hairpin RNA
TGF‐β1: Transforming growth factor‐β1
α‐SMA: Alpha‐smooth muscle actin

## 1 Introduction

Liver fibrosis is a progressive condition resulting from chronic liver damage, featured excessive accumulation of extracellular matrix (ECM), which can develop into cirrhosis, hepatocellular carcinoma, and liver failure [1]. Recently, several therapies for metabolic dysfunction‐associated steatohepatitis with liver fibrosis have emerged, including resmetirom and semaglutide [2]. However, specific drugs for advanced fibrosis or decompensated cirrhosis remain in preclinical development or clinical trials, highlighting the unmet medical needs in this field [3]. Hepatic stellate cells (HSCs), accounting for approximately 15% of the non‐parenchymal cells, have been identified as the primary effector cells of fibrosis [4]. Activation of HSCs induced by liver injury or microenvironmental stimuli leads to the secretion of large amounts of ECM molecules, ultimately driving liver fibrosis. Xu et al. found that brain and muscle arnt‐like protein‐1 (Bmal1) attenuates HSC activation during liver fibrosis by targeting glycolytic metabolism, a process mechanistically linked to the isocitrate dehydrogenase 1/α‐ketoglutarate (IDH1/α‐KG) axis under transforming growth factor‐β1 (TGF‐β1) stimulation [5]. A report by Zhi et al. revealed that carbon tetrachloride (CCl_4_) exposure or TGF‐β1 stimulation significantly upregulates G‐protein‐coupled receptor 81 (GPR81) expression, and knockdown of GPR81 inhibits CCl_4_‐induced liver fibrogenesis and TGF‐β1‐induced HSC activation [6]. Therefore, inhibiting the activated phenotype of HSCs has become an important strategy for treating liver fibrosis.

Accumulating evidence revealed that metabolic reprogramming is a central hallmark of the activation process of HSCs, with particularly prominent dysregulation of aerobic glycolysis [7]. Activated HSCs use aerobic glycolysis as a metabolic substrate to maintain cell proliferation, enhance ECM production, and facilitate migration to sites of hepatic injury [8]. Furthermore, dysregulated energy metabolism contributes to the progression of liver fibrosis and cirrhosis, highlighting the critical role of metabolic reprogramming in driving fibrogenesis [9]. Nevertheless, the underlying regulatory mechanisms of glycolysis in the activation of HSCs remain incompletely elucidated, and its regulatory network may involve the synergistic effects of multiple factors. Previous evidence has shown that selective inhibition of key glycolytic enzymes effectively inhibits HSC activation and ameliorates experimental liver fibrosis [10]. Jiang et al. reported that interleukin (IL)−17A promoted glycolysis and fibrosis of HSCs, which was abrogated by 2‐deoxy‐d‐glucose (2‐DG) [11]. Therefore, targeting aerobic glycolysis represents a promising therapeutic strategy to mitigate HSC activation and attenuate liver fibrosis. The S100 family, as a class of calcium‐binding proteins, plays a significant role in the fibrotic pathological processes of multiple organs, including the liver [12], kidney [13], and lung [14]. To date, twenty‐five members of the S100 family have been identified [15]. Among them, S100 calcium‐binding protein 16 (S100A16), the most recently characterized member, has been associated with the progression of fibrotic diseases. Jin et al. revealed that the interaction between S100A16 and GRP78 activates endoplasmic reticulum stress, contributing to renal tubulointerstitial fibrosis [16]. Previous studies have established a critical role for S100A16 in liver fibrosis. Zhang et al. demonstrated that S100A16 expression is markedly upregulated during HSC activation, and that genetic ablation of S100A16 effectively attenuates HSC activation both in vitro and in vivo, whereas S100A16 transgenic mice develop spontaneous liver fibrosis [17]. However, whether S100A16 mediates HSC activation through the glycolytic pathway and the potential regulatory mechanisms deserve further investigation.

Erb‐B2 receptor tyrosine kinase 2 (ERBB2) is a key member of the epidermal growth factor receptor (EGFR) family, characterized by an extracellular ligand‐binding domain, a single transmembrane helix, and an intracellular tyrosine kinase domain [18, 19]. Increasing researches have confirmed that ERBB2 can upregulate glycolytic marker proteins to participate in the glycolysis procession. For instance, studies in breast cancer have shown that ERBB2 upregulates lactate dehydrogenase A (LDHA) to promote glycolytic metabolism [20]. Furthermore, upregulation of ERBB2 has been implicated in fibroblast proliferation, migration, and activation [21]. For instance, Semaphorin 3E/Plexin D1 axis has been shown to promote pulmonary fibrosis through ERBB2‐dependent fibroblast activation [22]. However, the molecular mechanism by which S100A16 modulates glycolysis through ERBB2 to promote HSC activation has not been elucidated. This research investigated the functional role of S100A16 in CCl_4_‐induced liver fibrosis mouse models and TGF‐β1‐induced human liver stellate cells (LX2). Our study identified a previously unrecognized S100A16/ERBB2/glycolysis signaling pathway during HSC activation, revealing a crucial mechanism of metabolic reprogramming in liver fibrosis.

## 2 Materials and Methods

### 2.1 Cell Cultivate and Treatment

HSCs (LX2; human, male, liver of origin), obtained from Procell (CL‐0560, Wuhan, China; RRID: CVCL_5792), were verified by mycoplasma contamination testing and STR authentication. The cells were cultureed in Dulbecco's modified Eagle's medium (DMEM; PM150210, Procell) supplemented with 10% fetal bovine serum (FBS; 164210, Procell) and 1% penicillin/streptomycin (PB180120, Procell) at 37°C in a humidified incubator with 5% CO_2_. LX2 cells are a widely used, well‐characterized in vitro model for studying HSC activation. These cells exhibit many of the key characteristics of activated HSCs, including increased expression of alpha‐smooth muscle actin (α‐SMA), collagen, and other fibrotic markers. TGF‐β1 is a potent pro‐fibrotic cytokine and a key driver of HSC activation in vitro. For TGF‐β1 stimulation, LX2 cells were incubated with 10 ng/mL human TGF‐β1 (PeproTech, #100‐21) for 24 h [23]. For the inhibition of glycolysis, cells were treated with 2‐DG (5 mM; MedChemExpress, HY‐13966) 30 min prior to TGF‐β1 stimulation [11, 24].

### 2.2 Cell Transfection

Short hairpin RNA targeting S100A16 (sh‐S100A16), ERBB2 (sh‐ERBB2), an S100A16 overexpression plasmid (oe‐S100A16), along with their respective negative controls (sh‐NC and oe‐NC) were purchased from GeneChem (Shanghai, China). For transfection, LX2 cells were plated onto 6‐well plates (5 × 10^5^ cells/well) and allowed to grow until 80% confluency. Transfection was carried out with 2 μg plasmid DNA or shRNA with Lipofectamine 3000 (L3000008, Invitrogen) based on the manufacturer's instructions. After 48 h, the efficiency of transfection was evaluated using quantitative real‐time PCR (qRT‐PCR) or Western blot. In addition, the target sequences for sh‐S100A16 and sh‐ERBB2 are listed as follows: sh‐S100A16: CGATGAGTACTGGACCTTGAT; sh‐ERBB2: TGTCAGTATCCAGGCTTTGTA.

### 2.3 qRT‐PCR

Total RNA was extracted from cells or hepatic tissues using RNA Extraction Kit (R1100, SolarBio, Beijing, China), followed by reverse transcription into cDNA via the cDNA Synthesis Kit (P118‐100, GeneBetter, Beijing, China) following the manufacturer's instructions. Real‐time qPCR was conducted with SYBR Green qPCR Mix (P600‐500, GeneBetter) on a QuantStudio 6 Flex System (Applied Biosystems), with β‐actin as the endogenous control. Relative mRNA expression was quantified using the 2^−ΔΔCt^ method. For each target gene, the mean value of the control group was determined, and all individual sample values were normalized to this control mean for final presentation.

All primer sequences are provided in Table 1.

**Table 1:** Primer sequences used in this study.

Gene	Forward primer (5′‐−3′)	Reverse primer (5′‐−3′)
S100A16	GCTCCAGAAAGAGCTGAACCAC	ATGCCGCCTATCAAGGTCCAGT
α‐SMA	CTATGCCTCTGGACGCACAACT	CAGATCCAGACGCATGATGGCA
Collagen 1	GATTCCCTGGACCTAAAGGTGC	AGCCTCTCCATCTTTGCCAGCA
Fibronectin	ACAACACCGAGGTGACTGAGAC	GGACACAACGATGCTTCCTGAG
ERBB2	GGAAGTACACGATGCGGAGACT	ACCTTCCTCAGCTCCGTCTCTT
β‐actin	CACCATTGGCAATGAGCGGTTC	AGGTCTTTGCGGATGTCCACGT

*Note:* S100A16, S100 calcium‐binding protein 16; α‐SMA, alpha‐smooth muscle actin; ERBB2, Erb‐B2 receptor tyrosine kinase 2.

### 2.4 Western Blot

Cells or liver tissues were lysed using radioimmunoprecipitation assay (RIPA) buffer (V900854, Sigma‐Aldrich). Protein concentrations were determined via a Bicinchoninic Acid Protein Assay Kit (KIT‐BCA01, Sino Biological, Beijing, China). Equal amount of protein quantities were subjected to sodium dodecyl sulfate‐polyacrylamide gel electrophoresis (SDS‐PAGE) and transferred to polyvinylidene difluoride (PVDF) membranes (Millipore). After blocking, membranes were probed with primary antibodies overnight at 4°C, including S100A16 (11456‐1‐AP, 1:2000, Proteintech, Wuhan, China), hexokinase II (HK2; 22029‐1‐AP, 1:1000, Proteintech), LDHA (19987‐1‐AP, 1:2000, Proteintech), α‐SMA (14395‐1‐AP, 1:5000, Proteintech), Collagen 1 (ab316222, 1:1000, Abcam), Fibronectin (ab268020, 1:1000, Abcam), ERBB2 (18299‐1‐AP, 1:2000, Proteintech), and β‐actin (20536‐1‐AP, 1:5000, Proteintech). Following incubation with secondary antibodies for 1 h at room temperature, the membranes were subjected to signal detection using an enhanced Chemiluminescence Kit (P0018S, Beyotime, Shanghai, China) and quantification was performed using Image J software. The relative protein expression level of each target protein was calculated as the ratio of its densitometric value to that of β‐actin (target protein/β‐actin), with β‐actin serving as the loading control.

### 2.5 Cell Proliferation Assay

LX2 cell proliferation was determined utilizing a Cell Counting Kit‐8 (CCK‐8, C0038, Beyotime). Briefly, the treated LX2 cells were plated in 96‐well plates at a density of 2 × 10^3^ cells/well and incubated overnight. Thereafter, 10 μL of CCK‐8 reagent was introduced into each well. After 2 h of incubation, the absorbance (OD, 450 nm) values were then quantified with a microplate reader (BioTek).

### 2.6 Immunofluorescence Staining

Immunofluorescence was performed to evaluate α‐SMA levels in LX2 cells. Following fixation with 4% paraformaldehyde and permeabilization with 0.5% Triton X‐100, the cells were blocked with 5% bovine serum albumin (BSA). Subsequently, the cells were incubated overnight at 4°C with primary antibodies against α‐SMA (14395‐1‐AP, 1:1000, Proteintech). After incubation with secondary antibodies and 4'−6‐diamidino‐2‐phenylindole (DAPI), images were acquired under a fluorescence microscopy (Zeiss, Germany).

### 2.7 Detection of Lactate, Glucose Uptake, and Extracellular Acidification Rate (ECAR)

Lactate concentrations in cell supernatants and mouse liver tissues were determined with a Lactate Assay Kit (E‐BC‐K044‐M, Elabscience, Wuhan, China) in accordance with the manufacturer's guidelines. Glucose uptake was detected using a commercial kit (K676‐100, Biovision). ECAR, an indicator of glycolytic activity, was assessed using the Agilent Seahorse XF Glycolytic Stress Assay Kit (103020‐100, Agilent Technologies). LX2 cells were plated in XF24 plates at a density of 8 × 10^3^ cells/well and incubated overnight. After washing twice with XF medium, the cells were equilibrated for 1 h at 37°C in a CO_2_‐free environment. ECAR measurements were performed using the Agilent Seahorse XF24 Extracellular Flux Analyzer.

### 2.8 Bioinformatics Analysis

Potential interacting proteins of S100A16 were predicted using the Pathway Commons databases (http://www.pathwaycommons.org/) [25] and IntAct databases (https://www.ebi.ac.uk/intact/) [26] in July 2025. For both databases, the search was restricted to Homo sapiens (human) proteins to ensure species relevance. The default search criteria of the database were directly applied, and proteins related to glycolysis or fibrosis that appeared at the intersection of the two databases were included in the final analysis.

### 2.9 Co‐Immunoprecipitation (Co‐IP) Assay

The interaction between S100A16 and ERBB2 was examined using an Antibody Cross‐linking IP/Co‐IP Kit (P2180S, Beyotime). Briefly, cell lysates were immunoprecipitated with anti‐S100A16 (11456‐1‐AP, Proteintech), anti‐ERBB2 (18299‐1‐AP, Proteintech), or normal IgG antibody (30000‐0‐AP, Proteintech) overnight at 4°C. Protein A/G Magnetic Beads were then added and incubated with the lysate‐antibody mixture for 1 h. After washing with coupling buffer, the immunocomplexes were eluted and subjected to Western blot analysis.

### 2.10 Animal Experiments

Twenty‐four male C57BL/6 J mice (6 weeks old, 18–22 g) were available from Beijing Vital River Laboratory Animal Technology (China). All animals were maintained under standard conditions (temperature: 20 ± 2°C; humidity: 55–60%; 12‐h light/dark cycle) with standard chow and water ad libitum. All animal experiments were authorized by the Institutional Animal Care and Use Committee of Liuzhou Workers' Hospital (The Fourth Affiliated Hospital of Guangxi Medical University) and complied with the Guide for the Care and Use of Laboratory Animals.

### 2.11 Animal Model Establishment and Treatment

The CCl_4_ model is a classic and extensively used in vivo model for inducing liver fibrosis in rodents. CCl_4_ administration leads to hepatocyte injury, inflammation, and subsequent HSC activation, resulting in the deposition of extracellular matrix and the development of fibrosis. This model closely mimics the key pathological features of chronic liver injury and fibrosis observed in human patients. The animals were randomly allocated into four groups (*n* = 6 per group): control, CCl_4_, CCl_4_ + AAV‐sh‐NC, and CCl_4_ + AAV‐sh‐S100A16. Mice in the CCl_4_ groups were injected intraperitoneally with CCl_4_ (#289116, Sigma‐Aldrich) at a dose of 1 mL/kg body weight, prepared as a 10% solution in olive oil, twice weekly for 4 weeks [5]. Mice in the control group received an equal volume of olive oil. Mice in CCl_4_+AAV‐sh‐NC group and CCl_4_+AAV‐sh‐S100A16 group received 100 μL/mouse of adeno‐associated viral vector serotype 8 (AAV8) encoding sh‐NC or sh‐S100A16 (1 × 10^12 ^v.g/mL, Hanbio Biotechnology, Shanghai, China) via the tail vein 1 week before CCl_4_ administration. Three days after the final injection, mice were anesthetized with sodium pentobarbital (50 mg/kg, intraperitoneal injection), and blood and liver tissues were collected for subsequent experiments. The liver/body weight ratio was counted and calculated, serum alanine aminotransferase (ALT) and aspartate aminotransferase (AST) levels were detected using corresponding kits (E‐BC‐K1300‐M and E‐BC‐K1301‐M; Elabscience).

### 2.12 Histological Examination

Liver tissues were fixed in 4% paraformaldehyde, embedded in paraffin, and sectioned at a thickness of 5 μm. Hematoxylin and Eosin (H&E) and Masson staining was performed according to H&E Staining Kit (C0105S, Beyotime) and Masson's Trichrome Staining Kit (C0189S, Beyotime), respectively. H&E staining was performed to assess liver pathological changes, and Masson's trichrome staining was applied for assessing collagen deposition. Sections were analyzed under an Olympus light microscope, and images were acquired for further evaluation.

### 2.13 Statistical Analysis

Data are presented as the mean ± standard deviation from at least three independent experiments. Statistical analysis was conducted with GraphPad Prism 9 software. Group comparisons were carried out by Student's t‐test or one‐way analysis of variance (ANOVA) followed by Tukey's post‐hoc test. *p* < 0.05 was considered statistically significant.

## 3 Results

### 3.1 S100A16 Mediates TGF‐β1‐induced Glycolytic Activation in HSCs

**Figure 1:**
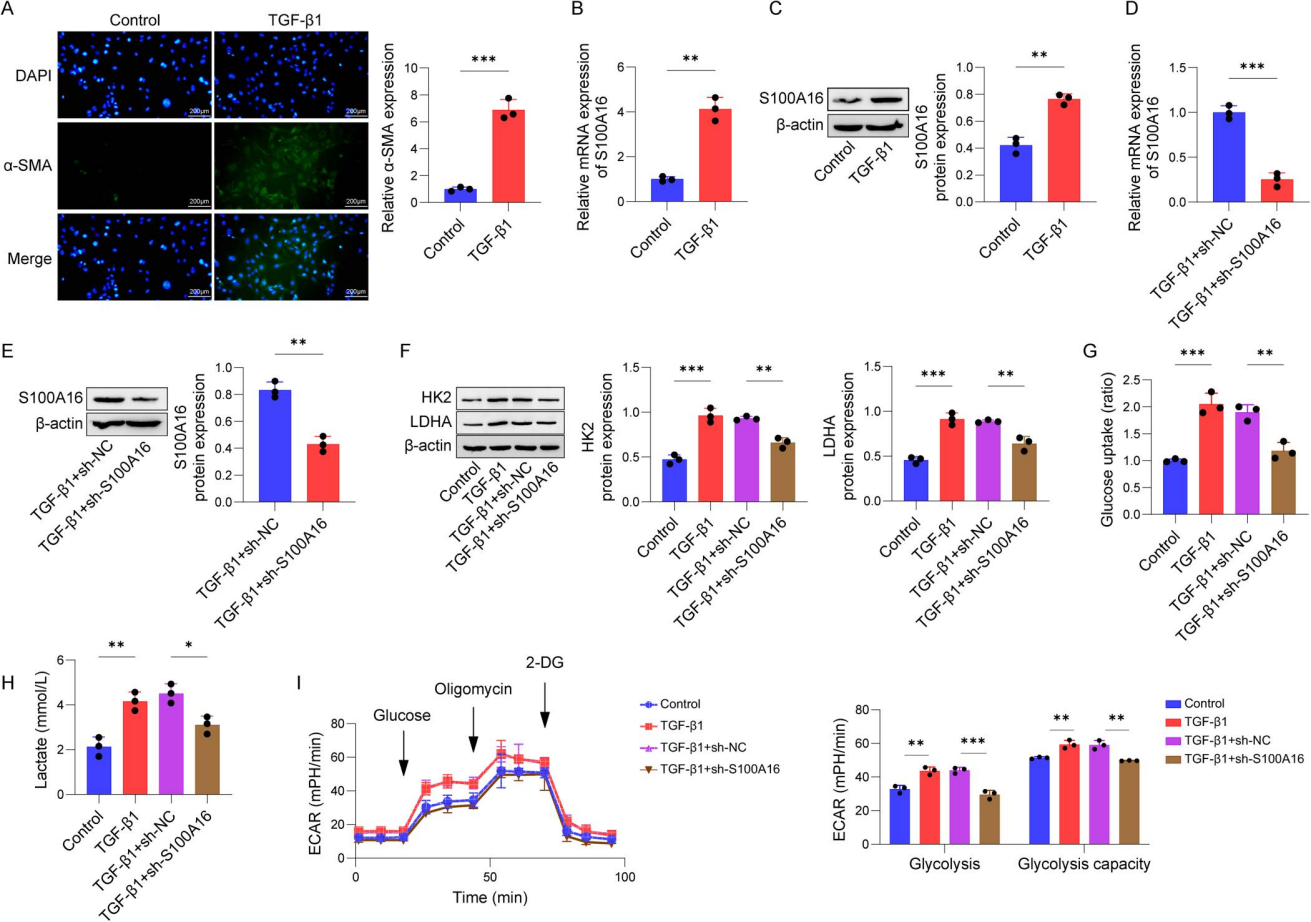
S100A16 knockdown inhibits glycolysis in HSCs. (A) α‐SMA protein expression in TGF‐β1‐induced HSCs was determined by immunofluorescence. (B) The mRNA level of S100A16 was detected by qRT‐PCR (C) Protein levels of S100A16 in LX2 cells induced by TGF‐β1. (D) qRT‐PCR and Western blot (E) were applied for determining S100A16 knockdown. (F) Western blot for HK2 and LDHA expression in LX2 cells treated with TGF‐β1. (G‐H) Extracellular lactate levels and glucose uptake in TGF‐β1‐treated LX2 cells after transfection of sh‐S100A16. (I) Seahorse glycolysis stress test measuring ECAR. n = 3, *p < 0.05, **p < 0.01, ***p < 0.001. S100A16, S100 calcium‐binding protein 16; α‐SMA, alpha‐smooth muscle actin; HSCs, hepatic stellate cells; TGF‐β1, transforming growth factor‐β1; qRT‐PCR, quantitative real‐time PCR; LX2, human liver stellate cells; HK2, hexokinase II; LDHA, lactate dehydrogenase A; ECAR, extracellular acidification rate.

Immunofluorescence staining (Figure 1A) revealed that TGF‐β1 significantly enhanced α‐SMA expression, confirming the activation of HSCs. qRT‐PCR and Western blot further demonstrated that TGF‐β1 stimulation markedly upregulated S100A16 mRNA and protein levels (Figure 1B–1C). To illustrate the function of S100A16 in regulating glycolysis in LX2 cells, we silenced S100A16 in TGF‐β1‐treated LX2 cells. Our data suggested that S100A16 knockdown notably reduced S100A16 mRNA and protein levels (Figure 1D–E). Additionally, silencing S100A16 significantly reduced the expression of key glycolytic enzymes, HK2 and LDHA (Figure 1F). TGF‐β1 treatment strongly increased glucose uptake (Figure 1G) and lactate production (Figure 1H) and elevated glycolysis and glycolysis capacity (Figure 1I), which were suppressed by S100A16 knockdown. Taken together, these findings suggested that S100A16 knockdown inhibits TGF‐β1‐induced glycolysis in HSCs.

### 3.2 Knockdown of S100A16 Inhibits HSC Activation Via the Glycolytic Pathway

**Figure 2:**
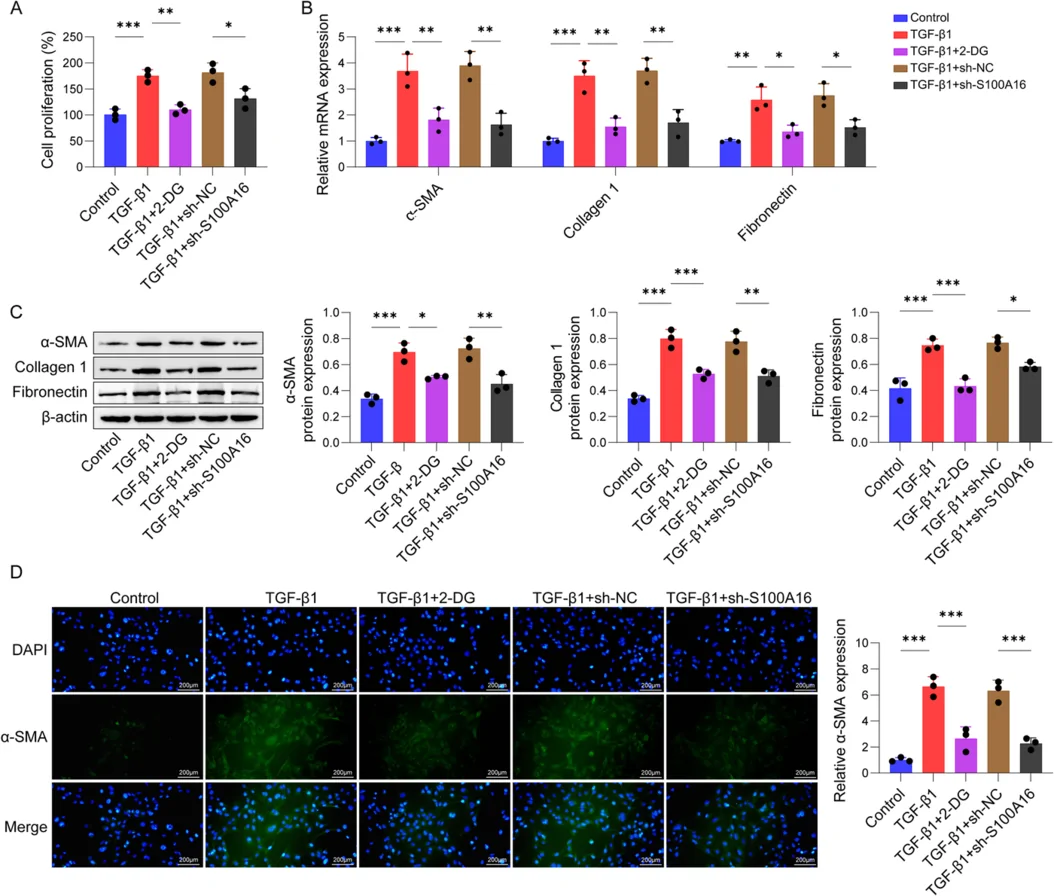
Knockdown of S100A16 inhibits activation of LX2 cells via glycolysis. (A) Cell proliferation of LX2 cells was assessed via CCK‐8 assay. (B) The mRNA levels of α‐SMA, Collagen 1, and Fibronectin was determined using qRT‐PCR. (C) The protein expression of fibrotic markers was detected by Western blot. (D) The protein levels of α‐SMA was measured by immunofluorescence. n = 3, *p < 0.05, **p < 0.01, ***p < 0.001. S100A16, S100 calcium‐binding protein 16; α‐SMA, alpha‐smooth muscle actin; LX2, human liver stellate cells; CCK‐8, Cell Counting Kit‐8; qRT‐PCR, quantitative real‐time PCR.

To further confirm whether S100A16 regulates HSC activation via glycolysis, sh‐S100A16 or 2‐DG, an inhibitor of glycolysis, was used to treat TGF‐β1‐induced LX2 cells. We found that TGF‐β1 treatment significantly increased the proliferation of HSCs, which was partially reversed by knockdown of S100A16 or glycolysis inhibition (Figure 2A). Next, we investigated whether glycolysis was associated with the severity of hepatic fibrosis. qRT‐PCR analysis showed that TGF‐β1 stimulation markedly upregulated the mRNA expression of key fibrotic markers, such as α‐SMA, Collagen 1, and Fibronectin. However, knockdown of S100A16 or glycolysis inhibition reduced the activation of LX2 fibrosis markers after TGF‐β1 treatment (Figure 2B). Consistent with these findings, Western blot analysis confirmed that TGF‐β1‐induced protein levels of fibrotic markers were dramatically reduced upon knockdown of S100A16 or glycolysis inhibition (Figure 2C). Immunofluorescence further demonstrated that TGF‐β1 significantly upregulated α‐SMA levels, and knockdown of S100A16 or inhibition of glycolysis decreased α‐SMA levels (Figure 2D). Furthermore, overexpression of S100A16 markedly aggravated TGF‐β1‐triggered HSC activation, as evidenced by upregulated α‐SMA and Collagen 1 protein levels. Notably, 2‐DG treatment largely abrogated the profibrotic effect induced by S100A16 overexpression, accompanied by obvious downregulation of α‐SMA and Collagen 1 (Figure S1). Collectively, these findings suggested that S100A16 promotes TGF‐β1‐induced HSC activation dependent on glycolytic metabolism.

### 3.3 S100A16 Interacts with ERBB2 to Promote Its Expression

**Figure 3:**
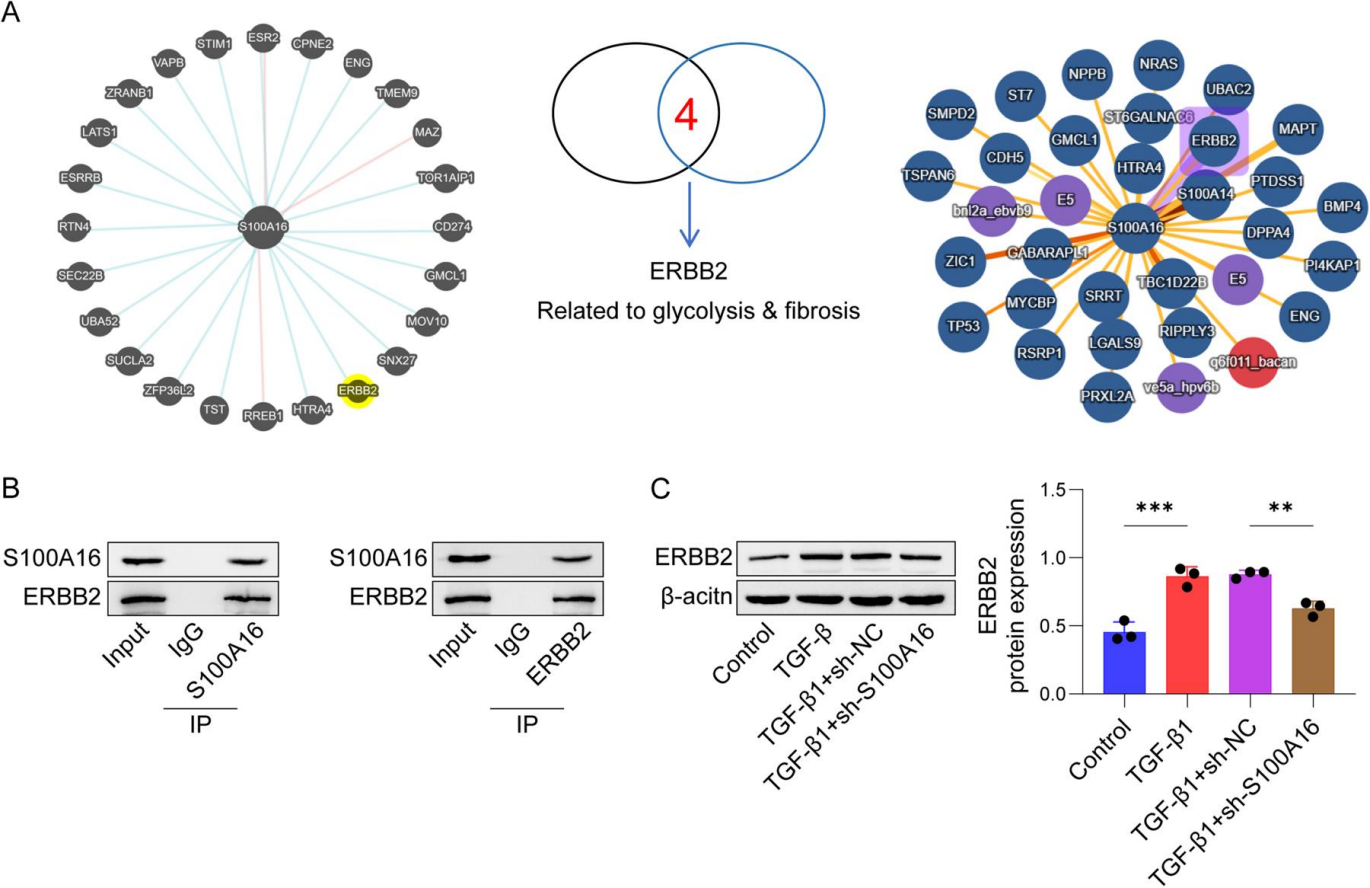
S100A16 interacts with ERBB2 in LX2 cells. (A) Pathway Commons and IntAct databases were applied for predicting the relationship between S100A16 and ERBB2. The left panel is derived from the Pathway Commons database, the right panel from the IntAct database, and the middle Venn diagram represents overlapping candidates between the two datasets. Data from both databases are used in accordance with their respective open‐access licenses (MIT license for Pathway Commons; CC BY 4.0 for IntAct), with proper attribution provided. (B) Co‐immunoprecipitation assay confirmed the binding between S100A16 and ERBB2. (C) The expression of ERBB2 was detected using Western blot. *n* = 3, **p < 0.01, ***p < 0.001. S100A16, S100 calcium‐binding protein 16; ERBB2, Erb‐B2 receptor tyrosine kinase 2; LX2, human liver stellate cells.

To elucidate the molecular mechanisms underlying S100A16‐regulated HSC activation, bioinformatics analysis was utilized to identify potential S100A16‐interacting proteins. Pathway Commons (left panel) and IntAct databases (right panel) predicted that ERBB2 (middle Venn diagram), a key regulator of glycolysis and fibrosis, might be a potential target for S100A16 binding (Figure 3A). Co‐IP confirmed that both S100A16 and ERBB2 could be detected in the IP complex, indicating that S100A16 physically combined to ERBB2 in LX2 cells (Figure 3B). Additionally, Western blot analysis showed that TGF‐β1 treatment upregulated ERBB2 levels. In contrast, silencing S100A16 significantly decreased ERBB2 protein levels relative to the sh‐NC group (Figure 3C). Moreover, knockdown of ERBB2 did not notably reduce the TGF‐β1‐induced increase in S100A16 protein level (Figure S2A). Overexpression of S100A16 further elevated TGF‐β1‐increased ERBB2 expression, which was significantly attenuated when ERBB2 was knocked down (Figure S2B), confirming that S100A16 functions upstream of ERBB2 in the TGF‐β1 signaling pathway in LX2 cells. These findings indicated that S100A16 binds to ERBB2 and enhances its expression, implying a key role of this interaction in HSC activation.

### 3.4 S100A16 Promotes HSC Activation through ERBB2‐mediated Glycolysis

**Figure 4:**
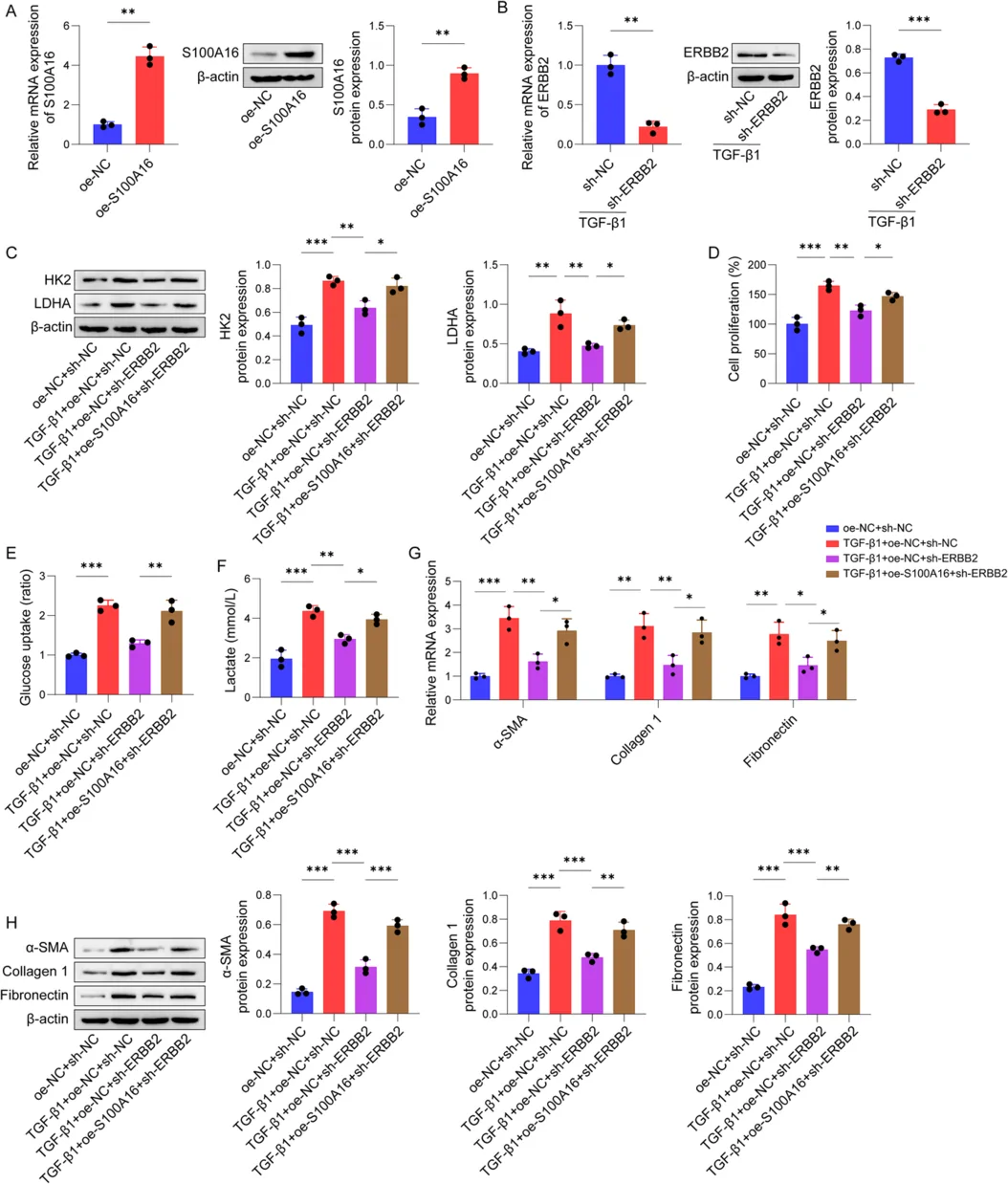
S100A16 promotes HSC activation by targeting ERBB2‐mediated glycolysis. (A) S100A16 expression in LX2 cells after transfection of oe‐S100A16. (B) ERBB2 expression in LX2 cells after transfection of sh‐ERBB2. (C) Western blot analysis was used to assess the expression of HK2 and LDHA in LX2 cells. (D) Cell proliferation was determined by CCK‐8 assay. (E‐F) Extracellular lactate levels and glucose uptake of LX2 cells after transfection of oe‐S100A16 and/or sh‐ERBB2. (G) mRNA expression of α‐SMA, Collagen 1 and Fibronectin was assessed by qRT‐PCR. (H) Protein levels of α‐SMA, Collagen 1 and Fibronectin was determined using Western blot. *n* = 3, *p < 0.05, **p < 0.01, ***p < 0.001. S100A16, S100 calcium‐binding protein 16; ERBB2, Erb‐B2 receptor tyrosine kinase 2; LX2, human liver stellate cells; sh, shRNA; HK2, hexokinase II; LDHA, lactate dehydrogenase A; CCK‐8, Cell Counting Kit‐8; qRT‐PCR, quantitative real‐time PCR; α‐SMA, alpha‐smooth muscle actin.

To further assess whether S100A16 promotes HSC activation by regulating glycolysis via ERBB2, we co‐transfected LX2 cells with oe‐S100A16 and/or sh‐ERBB2. qRT‐PCR and Western blot analysis confirmed that S100A16 overexpression significantly upregulated S100A16 mRNA and protein levels (Figure 4A), while ERBB2 knockdown markedly reduced ERBB2 mRNA and protein expression (Figure 4B). Next, we evaluated whether S100A16‐mediated glycolysis depends on ERBB2. We observed that ERBB2 inhibition downregulated TGF‐β1‐induced HK2 and LDHA protein levels (Figure 4C). Consistent with these findings, ERBB2 silencing significantly reduced TGF‐β1‐stimulated cell proliferation, glucose uptake, and lactate production (Figure 4D‐4F). Additionally, qRT‐PCR and Western blot analyses demonstrated that ERBB2 knockdown suppressed TGF‐β1‐induced expression of α‐SMA, Collagen 1, and Fibronectin (Figure 4G–4H). However, in cells overexpressing S100A16, the effects of sh‐ERBB2 on these markers were significantly attenuated. Further, knockdown of ERBB2 partially reversed S100A16 overexpression‐mediated HSC activation and reduced fibrotic marker expression (Figure S1). Together with the findings in Figure 3, which show S100A16 binds to ERBB2 and promotes its expression, these results indicated that S100A16 facilitates HSC activation through the ERBB2‐mediated glycolytic pathway.

### 3.5 S100A16 Promotes Liver Fibrosis through ERBB2‐mediated Glycolysis

**Figure 5:**
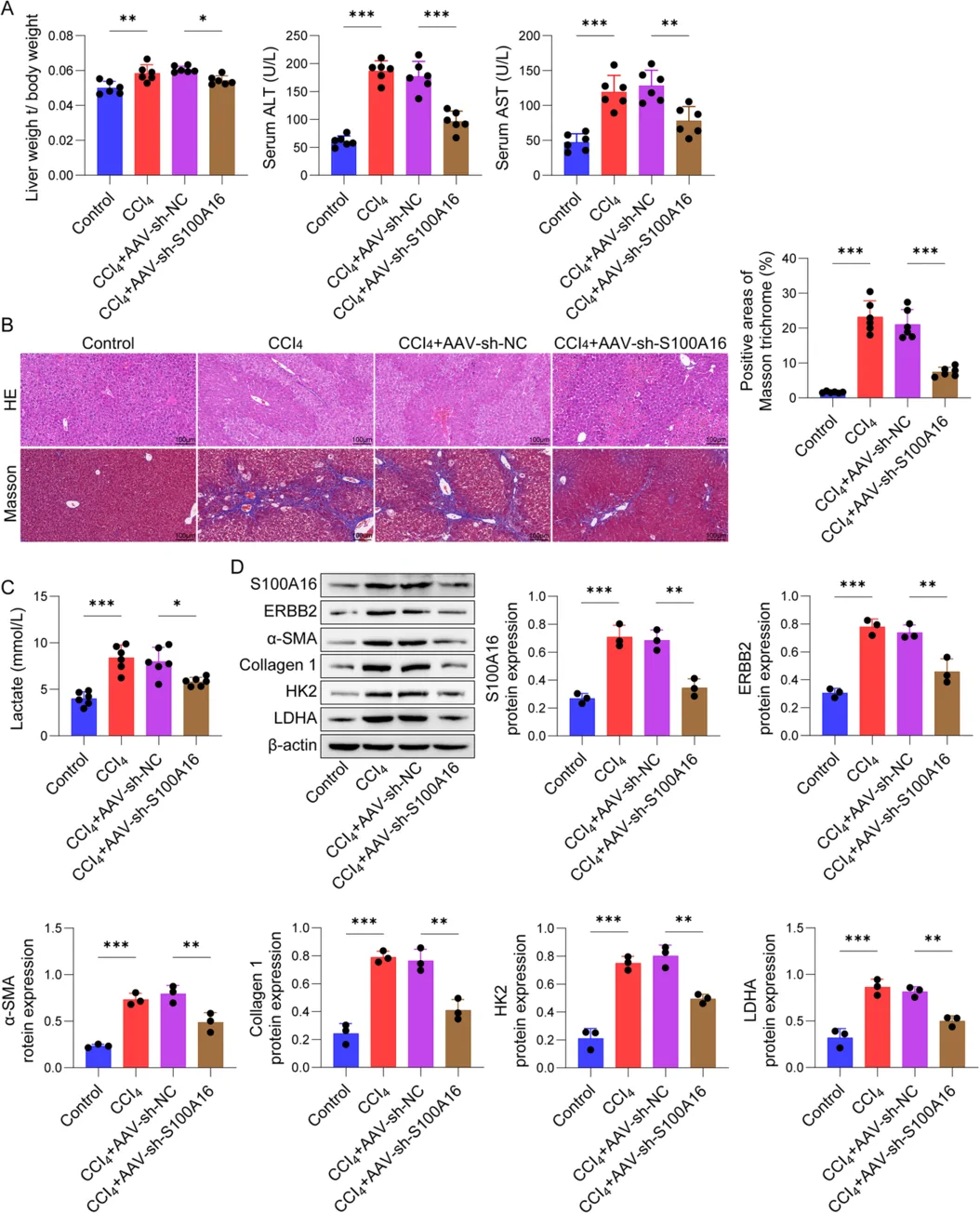
Knockdown of S100A16 alleviates liver fibrosis by inhibiting ERBB2‐mediated glycolysis. (A) Liver/body weight ratio, serum ALT level and AST level were determined. (B) Representative images of H&E and Masson staining in liver tissue sections of mice. (C) The liver lactate levels were measured by a commercial kit. *n* = 6. (D) The protein levels of S100A16, ERBB2, α‐SMA, Collagen 1, HK2 and LDHA in liver tissues were determined by Western blot. *n* = 3. *p < 0.05, **p < 0.01, ***p < 0.001. S100A16, S100 calcium‐binding protein 16; ERBB2, Erb‐B2 receptor tyrosine kinase 2; HK2, hexokinase II; LDHA, lactate dehydrogenase A; α‐SMA, alpha‐smooth muscle actin; H&E, hematoxylin and eosin; ALT, alanine aminotransferase; AST, aspartate aminotransferase.

**Figure 6:**
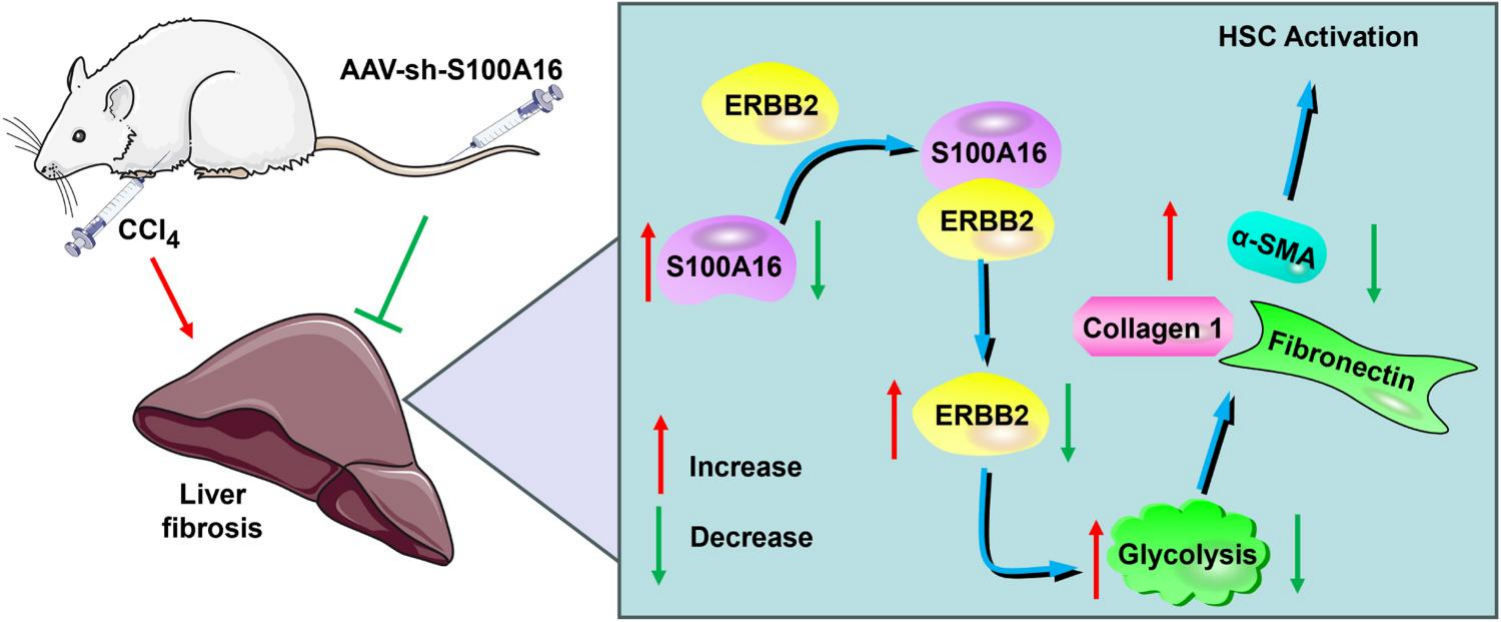
The schematic diagram shows the potential mechanism by which S100A16 promotes HSC activation and liver fibrosis through ERBB2‐mediated glycolysis.

To assess the therapeutic potential of targeting S100A16 in liver fibrosis, we investigated its effects on CCl_4_‐induced liver injury and fibrosis formation in mice. CCl_4_ exposure significantly increased liver/body weight ratio, serum ALT level and AST level, compared to the control group. However, AAV‐mediated S100A16 knockdown markedly alleviated these CCl_4_‐induced liver injury phenotypes compared with the sh‐NC group (Figure 5A). H&E staining and Masson's trichrome staining demonstrated that CCl_4_ injection remarkably promoted liver fibrosis compared to control mice, characterized by inflammatory cell infiltration, necrosis, and the formation of proliferating collagen fibers. S100A16 knockdown significantly alleviated CCl_4_‐induced hepatic cell necrosis and inflammatory cell infiltration. Additionally, collagen deposition was significantly reduced after S100A16 knockdown, indicating that S100A16 knockdown effectively attenuated CCl_4_‐induced liver fibrosis (Figure 5B). Furthermore, in liver tissues, S100A16 knockdown remarkably reduced the elevated lactate levels induced by CCl_4_ (Figure 5C). In line with the in vitro findings, S100A16 silence downregulated the expression of S100A16, ERBB2, α‐SMA, and Collagen 1 in CCl_4_‐induced mouse liver tissues (Figure 5D). Importantly, the CCl_4_‐induced upregulation of glycolytic enzymes HK2 and LDHA was also inhibited following S100A16 knockdown (Figure 5D). In summary, these findings suggest that S100A16 inhibition attenuates liver fibrosis by suppressing ERBB2‐mediated glycolysis. In conclusion, S100A16 binds to ERBB2 to upregulate glycolytic enzymes (HK2/LDHA), increasing lactate production and thereby activating HSCs, which accelerates liver fibrosis (Figure 6).

## 4 Discussion

Liver fibrosis is a progressive and difficult‐to‐treat pathological condition that may ultimately progress to cirrhosis or even liver cancer, but advanced fibrosis remains an area of high unmet medical need [27]. The key initiating factor of liver fibrosis is the activation of HSCs, and aerobic glycolysis is one of their primary metabolic characteristics. Previous studies have shown that HSC activation is associated with increased glycolytic activity [28]. Additionally, inhibiting key glycolytic enzymes can alleviate liver fibrosis in mice [29]. However, the precise molecular mechanisms by which glycolysis drives HSC activation and fibrosis formation remain incompletely understood. Our findings for the first time identify and validate the molecular cascade that S100A16 regulates glycolysis by interacting with ERBB2 to drive HSC activation, thereby providing a mechanistic link between S100A16, metabolic reprogramming, and liver fibrosis. These findings reveal that glycolysis is a key mechanism underlying S100A16‐mediated HSC activation, offering novel mechanistic insights into the development of liver fibrosis.

S100A16, as an important member of S100 family, has been found to exhibit significant differential expression in most human tumors [30]. Liver fibrosis, as a reparative response to liver injury, shares certain similarities with tumor development in its pathological characteristics, both characterized by rapid cell proliferation and abnormal angiogenesis. Among these, HSC activation is a central component of the fibrotic response following liver injury, with enhanced cell proliferation and migratory activity [31]. TGF‐β1, as a multifunctional cytokine, contributes significantly to liver fibrogenesis by activating HSCs. Zhang et al. identified homeobox C8 (HOXC8) as a pro‐fibrotic factor, whose expression is upregulated in both CCl_4_‐injured mouse livers and TGF‐β1‐stimulated HSCs, and demonstrated that its inhibition mitigates experimental fibrosis [32]. Clinical evidence from fibrotic liver patients also revealed a significant upregulation of S100A16 in HSCs, as reported by Zhang and colleagues [17]. Consistent with these studies, in this research, S100A16 was obviously elevated in TGF‐β1‐induced cells and CCl_4_‐induced mouse liver tissue, further confirmed that S100A16 is implicated in the pathological regulation of liver fibrosis. Additionally, TGF‐β1 regulates cellular function through metabolic reprogramming mechanisms, including enhancing aerobic glycolysis, promoting amino acid uptake, and increasing lactate production [33]. This study revealed that knockdown of S100A16 effectively inhibits TGF‐β1‐induced HSC glycolytic levels, suggesting that S100A16 may participate in HSC activation regulation by modulating the glycolytic pathway. Type I collagen, Fibronectin, and α‐SMA are key markers of activated HSCs [34]. Consistent with the findings of previous studies by Xu et al., this study confirmed that glycolysis inhibitor or knockdown of S100A16 can reverse the promoting effect of TGF‐β1 on HSC activation and the expression of these fibrosis markers [35]. Besides, S100A16 silencing significantly reduced the severity of CCl_4_‐treated liver fibrosis in mice and lowered serum lactate levels. These results corroborate the in vitro mechanistic studies, further confirming that S100A16‐mediated glycolytic regulation is critically involved in the pathogenesis of liver fibrosis. These findings provide new potential targets and theoretical basis for the prevention and therapy of liver fibrosis.

Protein‐protein interactions are central to many biological processes, and their dysfunction is associated with the pathogenesis of liver fibrosis [36]. In this study, we used the Pathway Commons and IntAct databases to predict proteins that interact with S100A16. The results showed that S100A16 interacts with ERBB2 and promotes ERBB2 expression. Mechanistically, previous research has confirmed that S100A16 stabilizes its target protein annexin A2 (ANXA2) by inhibiting ubiquitin‐mediated proteasomal degradation [37]. On the basis of this mechanism, we hypothesized that S100A16 binding to ERBB2 might similarly shield ERBB2 from E3 ligase‐mediated ubiquitination, thus stabilizing ERBB2 protein levels in HSCs. In addition, Yang et al. have reported that S100A16 can protect its interacting partners from K48‐linked polyubiquitination and proteasomal degradation through direct protein‐protein interactions [38]. Collectively, these findings support that S100A16 interacts with ERBB2 to maintain its protein stability, thereby promoting HSC activation. Consistently, our in vivo animal validation further clarified the upstream and downstream relationship of the S100A16/ERBB2 axis. In the CCl_4_‐induced liver fibrosis mouse model, AAV8‐mediated S100A16 knockdown markedly reduced hepatic ERBB2 expression, demonstrating that S100A16 functions as an upstream regulator to upregulate ERBB2 expression in liver fibrosis.

As a member of the EGF/ErbB family, ERBB2 is critically involved in cell growth and proliferation and has become a key regulator of glycolysis and fibrosis in various tissues. Zhou et al. demonstrated that ERBB2‐driven glycolysis is indispensable for breast cancer cell migration [39]. Additionally, Chen et al. further showed that ERBB2 upregulates LDHA, which in turn promotes breast cancer cell migration and invasion [40]. We validated the role of ERBB2 in HSC glycolysis, showing that knocking down ERBB2 downregulates TGF‐β1‐induced HK2 and LDHA protein expression. Moreover, ERBB2 activation participates in accelerating fibroblast activation, proliferation, migration, and the progression of pulmonary fibrosis [22]. In a renal fibrosis model, Li et al. found that upregulation of ERBB2 in tubular epithelial cells drives fibroblast activation and myofibroblast accumulation [41]. Notably, treatment with lapatinib, a dual ERBB1/ERBB2 inhibitor, has been shown to dose‐dependently suppress fibroblast activation markers and ameliorate skin fibrosis in vivo [21]. However, the role and mechanism of ERBB2 in liver fibrosis remain unclear. Our report is the first to demonstrate the involvement of ERBB2 in liver fibrosis. Our findings are consistent with these results, revealing that ERBB2 knockdown downregulates TGF‐β1‐induced cell proliferation, as well as α‐SMA, Collagen 1, and Fibronectin expression. Notably, S100A16 overexpression successfully rescued the glycolytic dysfunction and inactive phenotype of HSCs caused by ERBB2 deficiency, while ERBB2 depletion partially blunted the pro‐fibrotic and pro‐glycolytic effects of S100A16. These results confirm that S100A16 promotes HSC glycolysis and activation predominantly through an ERBB2‐dependent mechanism. Nevertheless, residual glycolytic and pro‐fibrotic activities persisted after ERBB2 knockdown, indicating that S100A16 also regulates HSC activation through ERBB2‐independent compensatory pathways. As a widely expressed oncogene, long‐term inhibition of ERBB2 may disrupt the homeostasis of normal tissues and trigger adverse reactions such as cardiotoxicity, thereby limiting its application in the clinical treatment of chronic liver disease [42]. In contrast, S100A16 is rarely expressed in normal liver tissue but is selectively upregulated during the fibrotic process. Therefore, S100A16 represents a safer and more specific therapeutic target for the treatment of liver fibrosis. Our study identified a novel S100A16/ERBB2/glycolysis signaling pathway during HSC activation, revealing a crucial mechanism of metabolic reprogramming in liver fibrosis.

Furthermore, several limitations of this study should be acknowledged. Firstly, the AAV8‐shRNA system used for in vivo knockdown mediates global hepatic silencing rather than HSC‐specific targeting, as AAV8 transduces multiple cell types within the liver. Therefore, we cannot exclude the possibility that S100A16 silencing in hepatocytes may alter the local metabolic microenvironment and indirectly modulate HSC activation and fibrogenesis, as discussed above. Future studies employing cell‐type‐specific conditional knockout mice are required to precisely define the HSC‐autonomous role of S100A16. Secondly, S100A16 may regulate additional metabolic pathways contributing to fibrogenesis in HSCs. Finally, the structural basis of the S100A16‐ERBB2 interaction requires further characterization to elucidate its mechanistic details. The clinical correlation between S100A16 and ERBB2 in liver fibrosis disease also needs to be validated. In summary, our study identifies S100A16 as a critical regulator of HSC activation through ERBB2‐dependent glycolytic reprogramming. We not only delineate a novel molecular pathway (Figure 6), but also pinpoint a potential therapeutic target for liver fibrosis. These findings significantly advance the understanding of metabolic regulation in fibrogenesis and may guide the development of mechanism‐based anti‐fibrotic strategies.

## Author Contributions

Shiyu Ou, Ling Du, and Zhongzhuan Li guaranteed the integrity of the entire study. Shiyu Ou, Shuwen Weng and Xiaoling Tang designed the study and literature research. Juan Liu, Jinping Qin and Zhongzhuan Li defined the intellectual content. Shiyu Ou, Xiaoling Tang, Rong Ouyang, and Shijiang Huang performed experiment. Shiyu Ou, Xiaoling Tang, Rong Ouyang, and Shijiang Huang collected the data. Xiaoling Tang, Juan Liu, Dingheng Liang and Shijiang Huang analyzed the data. Shiyu Ou, Xiaoling Tang, and Juan Liu wrote the main manuscript and prepared figures. All authors reviewed the manuscript.

## Ethics Statement

All animal experiments were authorized by the Institutional Animal Care and Use Committee of Liuzhou Workers' Hospital (The Fourth Affiliated Hospital of Guangxi Medical University) and complied with the Guide for the Care and Use of Laboratory Animals (Approval No. M202514).

## Conflicts of Interest

The authors declare no conflicts of interest.

## Declaration of Generative AI and AI‐Assisted Technologies in the Writing Process

During the preparation of this work the authors did not use any AI‐assisted technology.

## Supporting information


**Figure S1:** Inhibition of glycolysis or ERBB2 knockdown reversed S100A16 overexpression‐induced HSC activation.

**Figure S2:** S100A16 functions upstream of ERBB2 in TGF‐β1‐treated LX2 cells.

## Data Availability

The data that support the findings of this study are available from the corresponding author upon reasonable request.
